# Elaboration and validity of the *Instrumento para Avaliação de Modelos metodológicos voltados ao Desenvolvimento de Tecnologias*

**DOI:** 10.1590/0034-7167-2023-0046

**Published:** 2023-11-13

**Authors:** Cléton Salbego, Elisabeta Albertina Nietsche, Patrícia Bitencourt Toscani Greco, Nara Marilene Oliveira Girardon-Perlini, Silvana Bastos Cogo, Tierle Kosloski Ramos, Andrei Pompeu Antunes

**Affiliations:** I Universidade Federal de Santa Maria. Santa Maria, Rio Grande do Sul, Brazil; II Universidade Federal do Rio Grande. Rio Grande, Rio Grande do Sul, Brazil

**Keywords:** Psychometrics, Surveys and Questionnaires, Nursing Methodology Research, Technological Development, Nursing, Psicometría, Encuestas y Cuestionarios, Investigación Metodológica en Enfermería, Desarrollo Tecnológico, Enfermería, Psicometria, Inquéritos e Questionários, Pesquisa Metodológica em Enfermagem, Desenvolvimento Tecnológico, Enfermagem

## Abstract

**Objective:**

to elaborate and validate the Instrumento para Avaliação de Modelos metodológicos voltados ao Desenvolvimento de Tecnologias.

**Methods:**

a methodological study, developed in three stages: instrument structuring through documentary research and researchers’ expertise; instrument validity with a panel of 11 expert judge nurses; and instrument final composition.

**Results:**

the instrument, after validity by experts, consisted of 30 items, divided into the content (26 items) and appearance (four items) domains. In the initial instrument validity process, 11 items were modified and seven were withdrawn, as they had a percentage of agreement below 0.80. The instrument showed excellent internal consistency, with values greater than 0.90 in its psychometric criteria.

**Conclusion:**

the instrument produced and validated aimed at assessing research methodological models for technological development showed good reliability, and may contribute to the methodological rigor of technological development research in nursing.

## RESUMO

**Objetivo:**

elaborar e validar o Instrumento para Avaliação de Modelos metodológicos voltados ao Desenvolvimento de Tecnologias.

**Métodos:**

estudo metodológico, desenvolvido em três etapas: estruturação do instrumento por meio de pesquisa documental e da *expertise* dos pesquisadores; validação do instrumento com painel de 11 enfermeiros juízes especialistas; e composição final do instrumento.

**Resultados:**

o instrumento, após a validação por especialistas, foi composto por 30 itens, divididos entre os domínios conteúdo (26 itens) e aparência (quatro itens). No processo de validação do instrumento inicial, 11 itens sofreram modificação e sete foram retirados, pois apresentaram percentual de concordância inferior a 0,80. O instrumento apresentou ótima consistência interna, com valores superiores a 0,90 em seus critérios psicométricos.

**Conclusões:**

o instrumento produzido e validado voltado à avaliação de modelos metodológicos de pesquisa para o desenvolvimento tecnológico apresentou boa confiabilidade, podendo contribuir no rigor metodológico das pesquisas de desenvolvimento tecnológico em enfermagem.

## RESUMEN

**Objetivo:**

elaborar y validar el Instrumento para Avaliação de Modelos metodológicos voltados ao Desenvolvimento de Tecnologias.

**Métodos:**

estudio metodológico, desarrollado en tres etapas: estructuración de instrumentos a través de la investigación documental y la pericia de los investigadores; validación del instrumento con un panel de 11 enfermeros jueces expertos; y composición final del instrumento.

**Resultados:**

el instrumento, después de la validación por especialistas, constó de 30 ítems, divididos en los dominios contenido (26 ítems) y apariencia (cuatro ítems). En el proceso de validación del instrumento inicial, se modificaron 11 ítems y se retiraron siete, por tener un porcentaje de concordancia inferior a 0,80. El instrumento mostró una excelente consistencia interna, con valores superiores a 0,90 en sus criterios psicométricos.

**Conclusión:**

el instrumento producido y validado para evaluar modelos metodológicos de investigación para el desarrollo tecnológico mostró buena confiabilidad, y puede contribuir al rigor metodológico de la investigación en desarrollo tecnológico en enfermería.

## INTRODUCTION

Nursing has, over the years, demonstrated numerous efforts to meet the health needs of its patients in different social contexts, in order to become increasingly involved in care relationships. To follow the evolution of society in times of globalization and promote changes in care, management and teaching practices, nursing is dedicated to technological development^(1)^.

Technology production implies organizing a set of scientific and practical-everyday knowledge that, when systematized, helps in the “process of conception, elaboration, planning, ex-ecution/operationalization and maintenance of products and technological processes produced, validated and assessed by human beings, with specific practical purposes”(2:52). This context of technological production needs to be surrounded by a dynamic system of relationships (person-person, person-universe), allowing a creative process from dialogical, critical, reflective, ethical, social and transformative, individual and collective perspectives^(3)^.

Developing technologies in nursing comprises constructing, validating and assessing products and processes emerging from the understanding of human praxis, with a view to solving practical problems. It is characterized by the relationship between theory and practice and the interpretation and application of innovative propositions capable of contributing to social transformation^(3-4)^.

In order to reach valid, usual and effective tools for nursing work process’ emerging needs, researchers seek to innovate their studies using models and research methods built with a focus on the object of investigation. These methods comprise systematic structures for the logical and orderly planning of scientific research, allowing researchers to be instrumental in how to respond to the research object^(5)^. As for methodological models, they constitute representations or abstractions of what one wants to do, guiding the instantiation and sequence of what to do^(6)^. However, the empirical creation of methodological steps away from models or research methods that have already been validated and/or tested can bring important biases to investigations, compromising the results as well as scientific evidence quality.

Thus, it becomes important to insert in academic circles instruments that help in the internal validity of content and appearance of research models and methods with a focus on methodological development, allowing greater reliability and investigative validity. Thus, banalizing methodological adaptations are avoided and researchers’ time is optimized in conducting research.

Under this problem, self-created methodological models emerge for a given research that are anchored in using reliable, reliable and valid instruments. Based on this prerogative, it allows reducing the possibility of research biases, such as subjective judgments and mistaken inferences^(7)^. To this end, recognizing instrument quality guarantees the legitimacy and credibility of research results. Thus, creating a specific instrument to assess the methodological structure applied to health technology construction is a gap in health knowledge, especially in nursing, which daily demonstrates theoretical-practical evolution in technological production.

Considering this assumption, it is proposed, in this study, to develop a tool that allows researchers to analyze the methodological design applied to research to develop technologies, helping in its internal validity. There is a growing number of works available in the academic circles of nursing and health proposing technology construction, validity and/or assessment. They have used various research models, often seeking references from other areas^(8-9)^ to support them. However, technology production is observed as a gap in the knowledge produced by nursing, ignoring the concrete reality of a scenario/ population, using only elements available in the literature. Also, methodological designs adopted reveal theoretical, operational and analytical trends^(4)^.

Under this tension between attending to the advancement of science, developing technologies considering the target public’s praxis and offering greater reliability to adopted research designs, this research questioned: what items are necessary to compose an instrument for assessing methodological models built for technology development?

There is a lack of instruments in the scientific literature capable of assisting researchers in the validity of content and appearance of methodological models designed to conduct their research. Bearing this in mind, the construction and validity of a tool capable of subsidizing the methodological quality of technology development studies becomes feasible.

## OBJECTIVE

To develop and validate the *Instrumento para Avaliação de Modelos metodológicos voltados ao Desenvolvimento de Tecnologias* (IAMDT).

## METHODS

### Ethical aspects

The research is linked to a matrix project entitled “*Modelo Práxico para o Desenvolvimento de Tecnologias em Enfermagem: construção e validade na enfermagem*” . The study complied with ethical prerogatives involving research with human beings, obtaining approval by the Research Ethics Committee in 2021. Participating expert judges were informed about the objectives of the investigation and the nature of data collection. Those who agreed to participate signed the Informed Consent Form (ICF). Participant anonymity and confidentiality was guaranteed, and the possibility of withdrawal was informed, without any prejudice.

### Study design, period, and place

This is methodological research focused on the elaboration and validity of content of a measurement instrument for methodological models aimed at developing technologies. The results are organized based on the Guidelines for Reporting Reliability and Agreement Studies (GRRAS) precepts.

Production was guided by the following steps: (1) item structuring; (2) instrument content validity; (3) instrument final composition.

The instrument was built and validated between February and May 2022. Step two was performed remotely through Google Forms, WhatsApp and email to establish contact with expert judges.

### Population; inclusion and exclusion criteria

Eleven expert-judges participated in IAMDT content validity, meeting the following inclusion criteria: having a PhD in nursing; having carried out research on the development of technologies and/or measuring instruments; have guided research focused on these areas. Thus, six judges with expertise in developing technologies and five in the field of measuring instruments were included. The search was carried out by consulting the curricula available on the Higher Education Personnel Improvement Coordination (CAPES - *Coordenação de Aperfeiçoamento de Pessoal de Nível Superior*) *Lattes* Platform, publications in journals or by indication of experts.

### Study protocol

The instrument elaboration was conducted through three steps.

#### Step 1 – Instrument item structuring

This step was conducted through documentary research^(10)^ in the CAPES Catalog of Theses and Dissertations, with a view to mapping nursing theses and dissertations with a focus on technological development. The searches took place on the portal, using the terms “Nursing” AND “Technology” and applying the filters “master’s (dissertation)”, “doctoral (thesis)” and “nursing” as an area of knowledge, not establishing a time frame. The process was carried out in February 2020.

The study had the following question: what is the methodological systematic for the participatory development of technologies in nursing used in Brazilian theses and dissertations?

Theses and dissertations produced by nurses in Academic and Professional Graduate Programs, proposing technological development (construction, validity and/or assessment) based on emerging demands from the study context, i.e., proposals arising/thought/ prepared from of the target audience’s praxis, were included. Thus, those in which the technologies were produced on participatory principles were included. Dissertations and theses with incomplete abstracts, with a complete report not available and works that did not present the technology construction stage in detail, were excluded. In order to ensure the quality of this moment and avoid bias, study search and selection was carried out by two independent reviewers. The assessment of whether or not to include the study was based on reading titles and abstracts. For some cases it was necessary to access the full report. If there was disagreement between the reviewers, a third reviewer was involved.

The initial sample consisted of 1,729 publications. After reading the titles and abstracts, the sample was reduced to 410 studies. After reading the report in full, 337 did not respond to the research question. Finally, 73 reports were selected.

Data extraction was carried out with the help of Atlas.ti 9, considering descriptive variables of the studies and complementing them with data such as authorship, year of publication, Graduation Program of origin, type of technological production, study design, sample, data collection and data analysis technique(s), stages of technological development and its justification, type of technological production, application setting/technology use, purpose of technological production.

With this information in hand, a group of four nurses, two with expertise in technological development and two in instruments, analyzed each element with a view to constructing items that represented the technological construction process. These researchers are linked to research groups of the proposing educational institution, being intentionally selected. Thus, the group built 40 items in consensus.

#### Step 2 - Instrument content validity

After creating the items and domains for the IAMDT, content validity was carried out in the next step in order to verify with judges whether the proposed instrument had a relevant structure to assess research models capable of supporting technology construction.

For this step, a panel of 11 experts in the development of nursing technologies and health measurement instruments was created. PhD holders, with experience in the production or guidance of technologies or measuring instruments, carrying out research in this area, academic production in the area, were included. This information was consulted by accessing the researchers’ *Lattes Curriculum*.

To carry out validity, invitation letters were sent to experts, presenting the research objectives and explaining that participation in the study would occur by completing a characterization questionnaire, followed by the proposed items for validity of IAMDT content.

Upon acceptance, each judge received a questionnaire for analysis of the instrument, which was made available in an electronic form on Google docs. Item assessment considered the following psychometric criteria: objectivity (expressing desirability or preference), simplicity (expressing a single idea); clarity (being intelligible even to the lowest stratum of the population); relevance (being consistent with the attribute to be measured); accuracy (being distinct from the other items); modality (not using extreme expressions); typicality (using typical expressions for the attribute); and credibility (not appearing ridiculous, unreasonable or childish)^(11)^. Moreover, below each item, space was made available for experts to make suggestions or paraphrase the item according to the necessary adjustments, and the possibility of exclusion or permanence of items by judges was also considered. To analyze IAMDT content, the responses followed a Likert-type scale, with four levels: 1 (inadequate); 2 (little adequate); 3 (adequate); and 4 (completely adequate).

#### Step 3 – Instrument final composition

After the content validity step, changes were made to the items according to judges’ suggestions, and the updated version of the instrument was presented. The final version consisted of 30 items allocated in two domains (Content and Appearance).

### Analysis of result, and statistics

For analysis and organization, quantitative data were typed, through independent double typing, in a Microsoft Excel 2013 spreadsheet and, after checking inconsistencies, were submitted to statistical analysis using the Statistical Package for Social Sciences (SPSS) version 21. Items were described through absolute and relative frequencies. The Content Validity Index (CVI) was applied by adding the answers that scored three or four (adequate and totally adequate, respectively) on a Likert-type scale, assessing the degree of agreement among expert judges.

In content validity, the CVI of each item of the instrument was verified, assessing the degree of agreement among evaluators, with those in which CVI was equal to or greater than 0.80 being considered validated^(12)^. Those with a lower percentage were reformulated based on judges’ suggestions. These were analyzed by two researchers, aiming to avoid misunderstandings and/or inferences. Their suggestions contributed to the maintenance, modification, unification or removal of items. The instrument’s reliability was assessed using Cronbach’s alpha coefficient^(13)^.

The conduction of the study followed the GRRAS checklist prerogatives, which helps in the reliability of the presentation of results of agreement studies.

### RESULTS

The documentary study made it possible to define the theoretical/conceptual and operational content adopted for developing technologies in nursing, allowing the creation of two domains of assessment for the IAMDT, the content and appearance of a certain methodological model.

For IAMDT content validity, 29 judges were invited, and the instrument was submitted to a sample of 19 judges with a manifest interest in assessment. The instrument’s return rate was 57%, corresponding to 11 participants. All of them were nurses and, of these, nine (82%) were female and two (18%) were male, aged between 29 and 50 years. Training time ranged from five to more than 25 years. As for current professional occupation, it was found that all judges (100%) developed activities in higher education teaching, one (9.1%) of them maintaining care activities in parallel and one (9.1%) a health management position. All had PhD as their highest degree, and nine (82%) worked at public institutions and two (18%) at private institutions.

The IAMDT was initially built with 40 items arranged in two domains: 34 items in the content domain and six in the appearance domain. According to data shown in Table 1, all criteria had a CVI > 0.80.

**Table 1:** Content Validity Indexes of the *Instrumento e confiabilidade do Instrumento para Avaliação de Modelos metodológicos construídos para o Desenvolvimento de Tecnologias* based on psychometric criteria, Santa Maria, Rio Grande do Sul, Brazil, 2023

Psychometric criteria	CVI	Cronbach’s alpha
Objectivity	0.88	0.96
Simplicity	0.90	0.96
Clarity	0.85	0.96
Relevance	0.90	0.96
Accuracy	0.85	0.95
Modality	0.87	0.92
Typicality	0.88	0.92
Credibility	0.89	0.95

As for the reliability of the final version of the IAMDT, its internal consistency through Cronbach’s alpha coefficient, obtained values greater than 0.92 in the instrument’s eight psychometric criteria, demonstrating high internal consistency.

In terms of content validity, experts requested the exclusion of items 10, 19, 33, 39 and 40, where CVI ranged from 0.56 to 0.83. Items 1, 3, 6, 7, 8 and 24 were modified for clarity, with a CVI between 0.88 and 1.00. Items 12, 13, 14, 15, 29 and 30 were grouped into the other items, in order to minimize ambiguities, redundancy or repetitions, taking into account experts’ suggestions (Chart 1).

**Chart 1:** Changes made to items in the *Instrumento para Avaliação de Modelos/métodos construídos para o Desenvolvimento de Tecnologias*, Santa Maria, Rio Grande do Sul, Brazil, 2023

Items	Before judges’ assessment	CVI	After judges’ assessment
1	*O título do modelo/método representa seus objetivos?*	0.96	*O título representa seus objetivos?*
3	*Os conceitos representam os pressupostos do modelo/método?*	0.90	*Os conceitos expressam e representam os pressupostos do modelo/ método?*
6	*O nome de cada etapa/fase corresponde ao seu conteúdo?*	1.00	*O nome de cada etapa/fase está de acordo com o conteúdo apresentado?*
7	*Apresenta operacionalidade para a execução das suas etapas/ fases?*	0.88	*Apresenta os passos operacionais bem descritos para a execução de suas etapas/fases?*
8	*As fases/etapas se (inter)relacionam na busca da resolução do fenômeno?*	0.99	*As fases/etapas se (inter)relacionam na busca da representação do fenômeno?*
10	*Proporciona reflexões a respeito do tema?*	0.79	Item withdrawn. **Comments:** “I think it is not relevant to be on the instrument”; “I find this item too vague”.
13	*Incentiva a inserção do pesquisador no cenário da pesquisa?*	0.78	*Incentiva a participação ativa do(s) pesquisador(es) com o contexto da pesquisa?*
14	*Incentiva a participatividade do pesquisador com o cenário da pesquisa?*	0.63	
15	*Descreve a inserção ativa do pesquisador no campo de coleta?*	0.75	
19	*Contribui para o desenvolvimento de consciências (prática/ práxis)?*	0.74	Item withdrawn. **Comment:** “It is not clear what you want with this item here”.
24	*Incentiva a produção de conhecimento científico?*	0.93	Item withdrawn. **Comments:** “I think it’s similar to item 11, it can replace it.”; “Similar to question 11”.
29	*A linguagem é interativa, permitindo envolvimento participativo do(s)pesquisador(es)?*	0.82	*Sua linguagem interativa permite envolvimento participativo entre pesquisador(es) e pesquisado(s)?* **Comments:** “I think it could group researcher and researched, simplifying the item, considering that the participatory involvement of both is necessary”; “I think there are already questions about the interaction of those involved”.
12	*Apresenta linguagem adequada ao público-alvo?*	1,00	
30	*A linguagem é interativa, permitindo envolvimento participativo dos pesquisados?*	0.82	
33	*Facilita a obtenção de novos conhecimentos?*	0.83	Item withdrawn. **Comments:** “I don’t think it’s necessary. The important thing is to know if it generates new knowledge”; “I found it similar to items 11, 24, 25”.
39	*A proposta se caracteriza como um método de pesquisa?*	0.56	Items withdrawn.
40	*A proposta se caracteriza como um modelo metodológico para pesquisas?*	0.72	

After the content validity process, the IAMDT was produced in its final version containing 26 items in domain 1 (Content Validity) and four items in domain 2 (Appearance Validity) (Chart 2).

**Chart 2:** Validated final version of the *Instrumento para Avaliação de Modelos metodológicos voltados ao Desenvolvimento de Tecnologias*, Santa Maria, Rio Grande do Sul, Brazil, 2023

**1 *Discordo totalmente***	**2 *Discordo***	**3 *Discordo parcialmente***	**4 *Concordo***	**5 *Concordo totalmente***

*Assinale com um X a questão que melhor representa sua resposta*	1	2	3	4	5
***DOMÍNIO 1 - VALIDADE DO CONTEÚDO***					
*1. O título do modelo representa seus objetivos?*					
*2. O referencial teórico utilizado é pertinente e se aplica a proposta?*					
*3. Os conceitos expressam e representam os pressupostos do modelo?*					
*4. O modelo apresenta sistematicamente suas etapas/fases?*					
*5. O modelo apresenta clareza na descrição das etapas/fases?*					
*6. O nome de cada etapa/fase do modelo está de acordo com o conteúdo apresentado?*					
*7. O modelo apresenta os passos operacionais bem descritos para a execução de suas etapas/fases?*					
*8. As fases/etapas do modelo se (inter)relacionam na busca da representação do fenômeno?*					
*9. O modelo é adequado para interpretar a realidade prática?*					
*10. O modelo contribui para a construção do conhecimento na área?*					
*11. O modelo incentiva a participação ativa do(s) pesquisador(es) com o contexto da pesquisa?*					
*12. O modelo permite a (inter)relação pesquisador-pesquisado-contexto?*					
*13. O modelo auxilia o pesquisador na construção de hipóteses?*					
*14. O modelo fornece suporte metodológico e representacional ao desenvolvimento tecnológico?*					
*15. Sugere técnicas para estabelecer a comunicação e a cooperação para interpretar a realidade, levantar e priorizar os problemas e formular hipóteses?*					
*16. Incentiva a participação coletiva para a busca de soluções?*					
*17. A(s) técnicas de aproximação ao cenário da pesquisa incentivam uma ação participativa entre os envolvidos?*					
*18. Estabelece parceiros para a criação tecnológica, quanto a sua área de atuação e objetivos?*					
*19. O modelo representa o caminho para produção do saber técnico-científico?*					
*20. Apresenta sequência lógica das ideias, suas etapas/fases?*					
*21. As informações do modelo são claras, objetivas e representativas a proposta?*					
*22. O modelo incentiva a compreensão da realidade para poder contribuir na sua transformação?*					
*23. Sua linguagem interativa permite envolvimento participativo entre pesquisador(es) e pesquisado(s)?*					
*24. A linguagem do modelo está adequada para pesquisadores?*					
*25. Fornece elementos para o pesquisador realizar análises e sínteses sobre o objeto?*					
*26. Permite a descoberta, descrição, explicação, reprodução e controle de fenômenos, para o desenvolvimento de novos produtos e processos?*					
***Sugestões:***					
***DOMÍNIO 2 - VALIDADE DA APARÊNCIA***					
*27. As ilustrações do modelo (se houver) são claras e compreensíveis?*					
*28. As ilustrações do modelo (se houver) representam o conteúdo e operacionalidade das fases/etapas?*					
*29. As formas das ilustrações (se houver) estão adequadas a proposta?*					
*30. A disposição das figuras está coerente com o texto?*					
***Sugestões:***					

The total score must be calculated by adding the scores of the items, with a minimum of 30 and a maximum of 150 points. As for the score per domain, in domain 1 (Content), the minimum score is 26 and the maximum is 130 points. Considering domain 2 (Appearance), the score ranges from four to 20 points, the score ranges from four to 20 points. It is noteworthy that the higher the score, the more appropriate the model is.

### DISCUSSION

IAMDT elaboration and validity presents important elements to help researchers maintain the internal validity of their research with a focus on technological development. The instrument has innovative potential and contributes to constructing knowledge in nursing as it proposes theoretical and operational markers that can guarantee the methodological quality of studies aimed at creating technological tools in nursing.

Currently, there is a high number of publications on technologies, in which researchers empirically build their methodological structure in order to respond to the investigative object. This practice can expose the study to bias, which can impact technology usability for health.

A study’s quality assessment^(14)^ may vary in terms of its internal or external validity; however, the guarantee of this aspect will reflect on the reliability and trustworthiness of research results. In the scenario of technological construction, seeking strategies prioritizing methodological quality becomes an emerging factor with a view to presenting reliable evidence.

In the literature^(15)^, there are numerous valid and reliable tools that contribute to the quality of research, providing a high level of scientific rigor for technological development. As for an instance, the international literature points to important studies to support technological production, such as the Patient Education Materials Assessment Tool (PEMAT)^(16)^, used as a systematic instrument to assess and compare the comprehensibility and actionability of materials aimed at patients’ educational process in different contexts. Another instrument is the Suitability Assessment of Materials (SAM)^(17)^, a tool already validated for Portuguese^(18)^, which represents a systematic method to objectively assess the suitability of health information materials for patients.

In the Brazilian literature, we have highlighted so far two important tools aimed at qualifying studies of technological production^(19-20)^. The *Instrumento de Validade de Conteúdo Educativo em Saúde* (ICVES) ^(19)^ was built to meet the need to assess health products’ educational content, and it presented reliability through Intraclass Correlation Coefficient > 0.8. The other instrument used was the *Instrumento voltado para Validar Aparência de Tecnologia Educacional em Saúde* (IVATES)^(20)^, assessed with an overall CVI of the instrument equal to 0.93.

As for the IAMDT, according to the instrument’s overview, 83% items were assessed as excellent, with a CVI > 0.80. Therefore, it is possible to infer that the tool is valid to guide the design of research in technological construction. Considering the items in the content domain, the CVI of most items (n=22) was ≥ 0.80. Items on systematic presentation of stages, naming of each stage and interactive language to encourage participatory involvement between researcher and researched obtained a CVI equal to 1. These results point to the need for methodological research models with clear and coherent nomenclatures as well as adequate and assertive language for the actors involved to interrelate in a participatory, effective and constructive way.

The researcher-researched relationship is linked to dialogues based on causation, in order to reflect together on a certain fact/ object of investigation, with a view to thinking about a solution/ intervention capable of modifying a given reality. The real intention of interactive language with a causal focus must be clear and precise for producing research data to support robust interpretations and analyses, proposing results for daily practical needs^(21)^.

The IAMDT also aimed at assessing public participation in conducting research in technological development. The study population’s involvement in the process of creating health tools is increasingly highlighted^(22)^. Through this initiative, it becomes possible to incorporate individual or collective experiences of social actors in research’s activities and structures^(23)^. This integration is considered the main feature of participatory research, providing people with a voice to decide what is best for themselves^(23-24)^. In the IAMDT, five items in the content validity domain are linked to the assessment of participatory interaction between researcher, research setting and target audience. Items 9, 16 and 22 presented CVI ≥ 0.93, not requiring reformulations. As for item 11, its CVI was 0.63, being modified, at experts’ request based on suggestions (Chart 1), and maintained in the instrument.

Theory applied to technological development is another evaluative element in the IAMDT. A study^(25)^ focused on technology implementation describes that the guarantee of effective results of products and processes in health practice lies in the development, testing and refinement of theories on how their delivery can be optimized in the different contexts where it is applied. Theory can refer to proposed hypotheses and/or explanations of how it is expected that latent information from a research scenario and local actors interact with each other to propose or support technology production capable of provoking changes in the practical scenarios to which they are intended. In the IAMDT, items 2 (0.97), 3 (0.90), 13 (0.98) and 15 (0.87), directly linked to the need for structured models with strong theoretical bases, obtained satisfactory validity rates. However, items 13 and 15 were grouped together with 14, in order to contemplate the instrument’s objectivity.

Regarding the appearance validity domain in methodological models to develop technologies, CVI varied between 0.84 and 0.96 (items 28 and 27 respectively). Appearance validity^(20)^ aims to analyze the aesthetic approach attributed to tools containing lines, shapes, colors and image movement. Images incorporated in technological production must be harmoniously interrelated with the proposed tool’s content. Authors^(20,26)^ promote that apparent validity contributes to improving images and layout of a given product, facilitating the understanding of the proposed technology’s content. Illustrations have the potential to attract and convince readers, catching their attention, stimulating feelings and guiding the reader on the paths proposed in the study as well as presenting messages or a synthesis of knowledge.

The results obtained by validity with experts were important to qualify the instrument, helping researchers in the improvement of methodological models structured under a methodological script that will guarantee greater reliability to the study. Furthermore, as the IAMDT is used and assessed in research, it can be modified in order to meet different research needs in the technological context of nursing and health. Likewise, it will be possible to expand the psychometric tests of this instrument.

### Study limitations

As a limitation of this study, we highlighted the performance of only one round of assessment, suggesting the need to return it

to revise its structure. Another limitation is the lack of instruments with the same scope, which would allow a comparative study to be carried out, or even the comparison of results.

### Contributions to nursing and health

The work presents contributions to nursing in terms of carrying out research focused on technology construction, as the instrument developed is relevant and can contribute as a tool capable of guiding researchers in participatory technological development. The instrument will contribute as a guiding checklist, containing a methodological step-by-step for constructing/ developing technologies in nursing and health.

## CONCLUSIONS

The IAMDT represents an innovative tool to assist in the internal validity of methodologies applied to technological research in nursing. The instrument was assessed with good psychometric parameters, considering the objectivity, simplicity, clarity, relevance, accuracy, modality, typicality and credibility criteria.

In this study, the instrument was characterized as valid and reliable to assess research models for technology production. Using the IAMDT will allow new interpretative possibilities and resolution of existing methodological biases in research with self-created stages/phases for technology production. It is expected, in further works, to assess the instrument’s efficiency and effectiveness in terms of the purpose for which it is intended, thus making it possible to continue the validity process.
